# Comparison of Hemodynamic Performance, Three-Dimensional Flow Fields, and Turbulence Levels for Three Different Heart Valves at Three Different Hemodynamic Conditions

**DOI:** 10.1007/s10439-024-03584-z

**Published:** 2024-09-17

**Authors:** Lorenzo Ferrari, Dominik Obrist

**Affiliations:** ARTORG Center for Biomedical Engineering Research, University of Bern, Bern, Switzerland

**Keywords:** Aortic valve replacement, Tomo-PIV, BHV, BMHV, TRIFLO

## Abstract

The hemodynamic performance of different prosthetic heart valves is difficult to compare among studies due to a variety of test conditions and experimental techniques. Existing studies are typically limited to one family of valves (biological or mechanical) and testing conditions of 5l/min and often lack sufficient spatial resolution. To address these limitations, a pulse duplicator with a multi-view imaging system (Tomo-PIV) was employed to investigate the three-dimensional flow field in the aortic root of three different valves: a tri-leaflet mechanical heart valve (TRIFLO, Novostia), a bi-leaflet mechanical heart valve (On-X, Artivion), and a biological heart valve (Perimount, Edwards Lifesciences). The valves were tested at low (3 l/min), normal (5 l/min), and elevated (7 l/min) cardiac output $(CO)$ under hypotensive (40/60mmHg), normotensive (80/120mmHg), and moderate hypertensive (105/170mmHg) pressure conditions, respectively. Compared to the Perimount, peak mean velocity was − 33%, − 24%, − 18% for the TRIFLO and − 32%, − 20%, − 11% for the On-X at low, moderate, and elevated $CO$, respectively. Corresponding peak $TKE$ values decreased by − 66%, − 57%, − 44% (TRIFLO) and − 60%, − 50%, − 36% (On-X). At low $CO$, $EOA$ was lower for Perimount (1.07cm^2^) than for TRIFLO (1.47cm^2^) and On-X (1.52cm^2^), while it increased for elevated $CO$ to 2.75cm^2^ (TRIFLO) and 2.16cm^2^ (Perimount and On-X). For all valves, increasing $CO$ led to increased flow velocities, higher $EOA,$ and higher levels of turbulence, and the spatial influence of the valve on the flow field in the ascending aorta was extended. $TKE$ peaked closer to the STJ than for TRIFLO and Perimount.

**Supplementary Information:**

The online version contains supplementary material available at 10.1007/s10439-024-03584-z.

## Introduction

Aortic valve replacement is an established treatment option for severe valvular heart disease with more than 200,000 replacements performed annually worldwide [1, 2]. Commercially available surgical valves are mainly divided into the families of Mechanical Heart Valves (MHVs) and Biological Heart Valves (BHVs). The MHVs present a long-term solution associated with life-time anticoagulation therapy due to their thrombogenic potential, while BHVs offer better hemodynamic conditions and eliminate the need of anticoagulant therapy with the drawback of reduced durability [2–5]. The question on how to realize a durable valve replacement which does not require anticoagulation remains open. The assessment of flow characteristics downstream of these valves plays a significant role in understanding their acute and long-term performance, optimizing their design, and supporting the choice of the most suitable replacement. To this end, several experimental as well as computational investigations were carried out in the past years (e.g., 6–9). These studies comprehensively investigated the flow characteristics downstream of a surgical valve targeting an ideal condition of a normotensive patient with a cardiac output $(CO)$ of 5l/min. However, patient flow conditions can differ from the idealized conditions of these experiments so that recent standards require valve validation at reduced and increased values of $CO$ [10–12]. In addition, the comparison of hemodynamic results from different studies on surgical valves remains challenging and inconsistent due to differences in manufacturers’ size labeling, techniques of investigation, and testing conditions [13–16]. To date, comparative studies of bioprosthetic [17–19] or mechanical replacement [20, 21] are restricted to one category of valve, mostly analyzing the transvalvular pressure gradient and orifice area and are often limited by coarse structural or temporal resolution.

To address these limitations, this study aims to provide a comprehensive comparison of the hemodynamic performance parameters, the mean velocity fields, and the turbulence levels for three different valve types under three different flow and pressure conditions. It uses a validated in vitro Tomographic Particle Image Velocimetry (Tomo-PIV) system to measure the flow field in the aortic root [9, 22]. Tomo-PIV is a widely used technique in experimental fluid mechanics which allows for the quantification of all three velocity components in the three-dimensional flow domain. Such measurements are essential when complex flows without inherent symmetries or turbulent flows are investigated [23]. Therefore, three-dimensional experimental methods are increasingly used to study the complex flow field past prosthetic heart valves [24–27].

## Material and Methods

A multi-view imaging system for Tomo-PIV was employed to assess the three-dimensional flow field in the aortic root with three different aortic valve prostheses at three different hemodynamic conditions. The experimental setup was previously used to study surgical and transcatheter heart valves [9, 22, 26] and was further adapted to perform tests at low and elevated $CO$.

### Test System

**Fig. 1:**
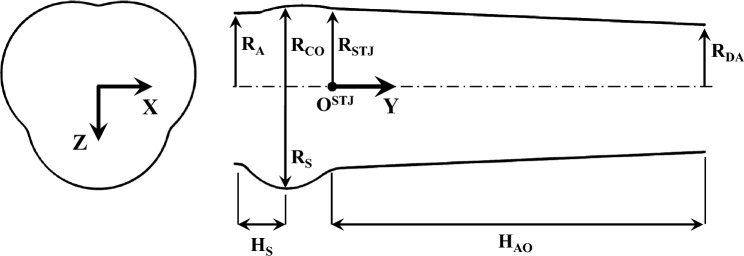
Lumen geometry of the silicone phantom mimicking the aortic root with reference to the main geometrical parameters of Table 1.

The tested valves were tightly mounted in a thick-walled silicone phantom (SP) featuring an idealized aortic root geometry. The silicone phantom had an annulus diameter of ${D_{\text{a}}} = 26{\text{mm}}$ three hemispherical bulges mimicking the sinuses of Valsalva, and a straight ascending aorta with a decreasing diameter from 27.5mm in the sino-tubular junction (STJ) to 22mm distal diameter [28]. Details of the aortic root geometry are given in Figure 1 and Table 1. The silicone phantom and the aortic valve prothesis (AVP) were integrated in a pulse duplicator which reproduced physiological pulsatile flow using a computer controlled volumetric pump (VP). During the systolic phase, the forward movement of the VP’s piston compressed a silicone model of the left ventricle (LV) and directed the fluid toward the test section.

**Table 1:** Relevant geometrical parameters of the aortic root silicone phantom.

Parameter	Value [mm]	Description
H_s_	7.70	Sinus half-height
H_AO_	63.50	Ascending aorta height
R_A_	13.00	Aortic annulus radius
R_S_	17.055	Sinus radius
R_CO_	13.75	Commissure radius
R_STJ_	13.75	Ascending aorta radius
R_DA_	11.00	Distal aorta radius

**Fig. 2:**
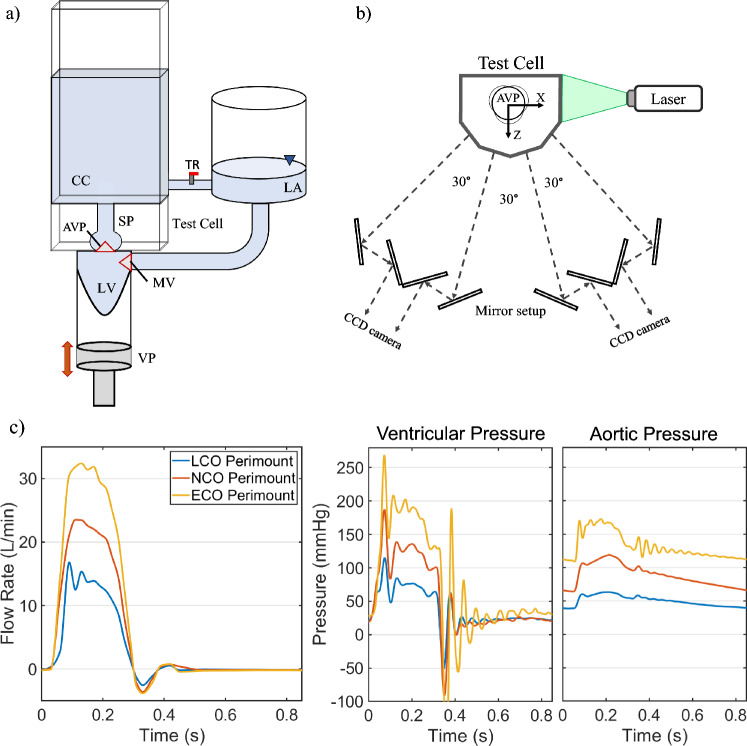
**A** Schematic of the pulse duplicator. **b** Top view on mirror setup for multi-view image acquisition. Four different angles are captured simultaneously by the camera by splitting each field of view in half. **c** Example of ensemble-averaged flow rate (left) and instantaneous pressures (right) for the Perimount valve at each condition. These curves are also representative for the waveforms of the On-X and TRIFLO valves.

After the test section, the fluid passed through a compliance chamber (CC) and a tuneable resistor (TR), which allowed the tuning to different pressure conditions. It was then collected in an open chamber mimicking the left atrium (LA). The backward movement of the piston initiated the diastolic phase, during which the LV expanded and filled with fluid from the LA. To prevent backflow from the LV to the LA, these two chambers were separated by a bi-leaflet mechanical heart valve of 25mm diameter (On-X, Artivion) which modeled the action of the mitral valve (MV). A schematic of the pulse duplicator is shown in Figure 2a and further details can be found in [29].

Pressure values were monitored and recorded with disposable pressure transducers (Xtrans, Codan, Germany) in the LV and the CC, immediately upstream of the valve and downstream of the SP, respectively. Pressure values were acquired at 1000Hz and a total of 60 consecutive beats were captured for further analysis. The flow rate was monitored with an ultrasonic flow probe (Transonic Systems Inc., Ithaca, NY, USA) placed between the CC and the TR.

A Refractive Index-Matched (RIM) fluid with a density of ${{{\rho }}_\text{f}}{\text{ = 1,200kg/}}{{\text{m}}^{\text{3}}}$ and a kinematic viscosity $\nu  = 4.7{\text{m}}{{\text{m}}^2}{\text{/s}}$ was used under a controlled temperature of 23°C. The test fluid was obtained by mixing a solution of water/glycerol/NaCl (Sigma Aldrich Corporation, St. Louis, MO, USA), with a respective weight ratio of 0.494:0.340:0.166.

### Tomo-PIV Measurement System

Four images of the particle-seeded fluid were acquired simultaneously using a mirror setup and 8M pixel CCD cameras (Imager LX, LaVision GmbH, Göttingen, Germany) as shown in Figure 2b. The prime lenses mounted on the cameras had a focal length of $f = 100{\text{mm}}$ and a maximum aperture of F2.8 (Kenko Tokina, Tokyo, Japan). The final resolution of each recorded image corresponded to 1656 × 2488 pixels. The particles used as passive tracers were PMMA based (density ${{{\rho }}_{\text{p}}}{\text{ = 1180kg/}}{{\text{m}}^{\text{3}}}$) with Rhodamine B coating (microparticles GmbH, Berlin, Germany) and a mean diameter of ${{d}_{\text{p}}} = 35{\mu\text{m}}$. These particles were excited with a dual cavity Nd:YAG laser (Nano L 200-15 PIV, Litron Systems Ltd, Rugby, UK) at a wavelength of $\lambda  = 532{\text{nm}}$. The width of the laser was set to 15ns and the interframe time between consecutive laser pulses was adjusted to ensure adequate particle displacement. During systole, the pulse delay $\Delta t$ was set to 300, 200, and 150μs for low, normal, and elevated $CO$, respectively. As lower velocities were expected during diastole, the pulse delay was extended to 800μs for all $CO$. To selectively collect the light emitted from the particle, cameras were equipped with a low-pass filter (570nm cut-off wavelength). Camera and volume calibration, images pre-processing, volume reconstruction, and the computation of the 3D instantaneous velocity vector fields with a 3D cross-correlation algorithm were performed with the software DaVis 10 (LaVision GmbH, Göttingen, Germany). The last step of the 3D cross-correlation routine determined the resolution ${\delta}$ of the experiment which was the same in all spatial directions $(\delta {\text{x}} = \delta {\text{y}} = \delta {\text{z}})$. With an interrogation volume of 48 voxels (1.71mm) and 75% correlation overlap, the final spatial resolution of the velocity field was

1 $$\delta = 48 \cdot (1 - 0.75) \cdot 1\text{vx }\approx 0.43\text{mm}.$$

Further details on the test system and the measurement system can be found in [9, 26].

The following valves were tested in a total of nine separate experiments:

- Bi-leaflet mechanical heart valve (On-X, Artivion).
- Tri-leaflet mechanical heart valve (TRIFLO, Novostia).
- Biological heart valve (Carpentier-Edwards Perimount, Edwards Lifesciences).

**Fig. 3:**
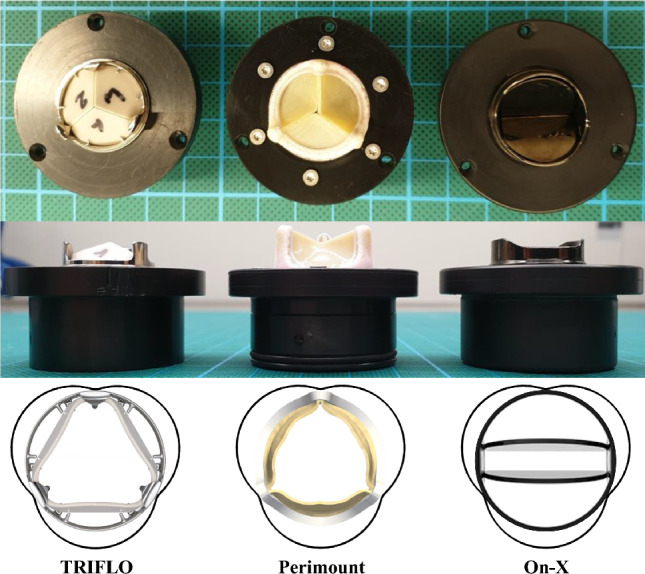
From top to bottom: top and side views of the mounted valves and the azimuthal orientation of TRIFLO, CEP, and On-X (left to right) with respect to the sinus portions. Valves on the bottom of the figure are represented in idealized open position.

All tested valves had a labeled diameter of 21mm, while the internal diameter according to manufacturer specifications corresponded to 19.4, 19.6 and 20mm for On-X, TRIFLO and Perimount, respectively. After removing the suturing ring, the mechanical valves were tightly inserted in the annulus position of the test section (Figure 3, top). For the Perimount, it was not possible to remove the suturing ring. Instead, it was fitted at the same height as the mechanical valves by clamping (Figure 3, middle).

The silicone phantom was placed on top of the valve so that the aortic annulus of the phantom was aligned with the valve, with the leaflets facing the sinuses. The On-X was aligned with one leaflet facing the front sinus (Figure 3, bottom).

Each valve was tested at low, normal and elevated $CO$ under hypotensive, normotensive and hypertensive pressure conditions, respectively (Table 2). In each of the nine experiments, the flow rate was prescribed by changing the settings for the piston pump and by adjusting the systemic compliance and resistance in the pulse duplicator. The pressures in the LV and the CC were used to compute the transvalvular pressure gradient.

**Table 2:** Experimental parameters. Testing parameters were selected according to ISO 5840-1:2021[10]

Mimicking Condition	Flow rate [l/min]	Aortic pressure [mmHg]	HR [bpm]	Systolic duration [%]	Re	Wo
Hypotensive	3	40-60	70	35	≈3500	≈13
Normotensive	5	80-120	70	35	≈5000	≈13
Hypertensive	7	105-170	70	35	≈7000	≈13

The Reynolds and Wormsley number were computed as follows:

2 $$Re=\frac{{4 Q}_{max}}{{D}_{a}\pi \nu },$$

3 $$Wo=\frac{{D}_{\text{a}}}{2}\sqrt{\frac{2\pi }{T\nu }},$$

where Qmax was the peak systolic flow rate and $CO$ was the diameter at the inlet of the valves.

Instantaneous flow fields were acquired for $N=200$ pulses at different phases $\phi$ of the cardiac cycle such that each image was acquired at time ${t_{\text{n}}}={{{t}}_{{\varphi }}} +  (n - 1)T$ where $n = 1,2, \ldots ,N$ and *T* is the period of the heart cycle.

### Data Analysis

The velocity field was separated according to the Reynolds decomposition into fluctuating ($v^{\prime}$) and mean ($\overline{v }$) components [30]:

4 $$v= \overline{v }+v{^\prime}.$$

The mean velocities $\overline{v} (x)$ were computed by phase averaging the instantaneous velocity field:

5 $$\overline{v }\left(\text{x},t_{{\varphi }}\right)= \frac{1}{N} \sum_{n=1}^{N}v(x,{t}_{{\varphi }}+(\text{n}-1)\text{T}).$$

A total of 25 phases ${t_{\phi}}$ were chosen with increasing density in proximity of the systolic peak: ${t_{\phi}}$ = [0.00; 0.03; 0.06; 0.09; 0.11; 0.13; 0.15; 0.17; 0.19; 0.21; 0.23; 0.25; 0.27; 0.29; 0.31; 0.33; 0.36; 0.39; 0.42; 0.45; 0.53; 0.61; 0.69; 0.77; 0.85] sec.

The mean flow rate was extracted from the reconstructed velocity profile as follows:

6 $$\overline{Q }={\overline{v} }_{y}\cdot A,$$

where $A$ is the cross-sectional area of the aorta and ${\overline{v} }_{y}$ the mean streamwise velocity. Flow rate values were computed at multiple streamwise locations (with a spacing of 0.4mm) between sino-tubular junction (STJ) and the distal end of the ascending aorta.

The effective orifice area $EOA$ was calculated from flow and pressure according to standard (ISO 5840-1) [10] as follows:

7 $$EOA=\frac{{Q}_{RMS}}{51.6\sqrt{\frac{{\Delta P}_{mean}}{\rho }}},$$

where ${Q_{{\text{RMS}}}}\;{\text{(ml/s)}}$ is the root-mean square of the flow computed during positive pressure difference period and $\Delta {P_{{\text{mean}}}}\;({\text{mmHg}})$ is the mean systolic transvalvular pressure gradient.

The turbulent kinetic energy $tke$ [J/kg] was computed from the three components of the velocity fluctuations $\left[{v}_{x}^{\prime},{v}_{y}^{\prime},{v}_{z}^{\prime}\right]$ as follows:

8 $$tke\,=\,\frac{1}{2}(\overline{{v }_{x}^{\prime}}\overline{{v }_{x}^{\prime}}+\overline{{v }_{y}^{\prime}}\overline{{v }_{y}^{\prime}} +\overline{{v }_{z}^{\prime}{v}_{z}^{\prime}}).$$

The total turbulent kinetic energy $TKE [J]$ and the planar turbulent kinetic energy, $planar TKE [J/m]$, were assessed by integration of $tke$ over the volume of the ascending aorta $(V)$ extending from the STJ to 60mm past the STJ, and the cross-sectional areas $(A)$ perpendicular to the streamwise direction, respectively:

9 $${TKE}={\rho}_{f}{\int }_{V}tke\, dV,$$

10 $${planar TKE}={\rho}_{f}{\int}_{A}tke\, dA.$$

The energy dissipation rate $\epsilon$ was calculated from the rate of strain tensor of the velocity fluctuations as follows:

11 $$\epsilon =2{\nu }_{f}\overline{{S }_{ij}{S}_{ij}},$$

where ${S_{ij}}$ is the symmetric component of the velocity fluctuation gradient tensor:

12 $${S}_{ij}= \frac{1}{2}\left(\frac{\partial {v}_{i}^{\prime}}{\partial {x}_{j}}+\frac{\partial {v}_{j}^{\prime}}{\partial {x}_{i}}\right)\, with\, i=x,y,z \,and \,j=x,y,z.$$

The Kolmogorov length scale $\eta$ was computed from the energy dissipation rate $\epsilon$ assuming homogeneity and local isotropy:

13 $$\eta ={\left(\frac{{\nu }^{3}}{\upepsilon }\right)}^\frac{1}{4}.$$

### Uncertainty and Convergence

The intrinsic uncertainty of PIV measurements can be split into systematic and random uncertainty [31]. The systematic uncertainty was minimized adopting the methodologies outlined by Hasler et al. (2016) and Hasler and Obrist (2018) [9, 22]. In addition, experiments were performed after obtaining a mean mapping error < 0.1 pixel during calibration with associated peak values < 0.2 pixel. In general, a Tomo-PIV reconstruction can be considered reliable for a mapping error < 0.4 pixel and considered to be of high quality for an error < 0.1 pixel [32]. The random uncertainty of the experiments was associated with the number of subsequent acquisitions $(N  =  200)$ which were analyzed. The random uncertainties of the mean streamwise velocity and $tke$ were estimated by [33]:

14 $${U}_{\overline{x} }={\sigma }_{x}\sqrt{\frac{1}{N}}, $$

15 $$ U_{{tke}}  = \sqrt {\left( {\overline{{v_{x}^{\prime2} }} } \right)^{2}  + \left( {\overline{{v_{y}^{{\prime}2} }} } \right)^{2}  + \left( {\overline{{v_{z}^{{\prime}2} }} } \right)^{2} } \sqrt {\frac{1}{{2N}}},$$

where ${\sigma }_{x}$ is the standard deviation of the velocity components in $x$-direction. These uncertainty measures were computed in the entire investigated domain and normalized with the respective peak value as follows:

16 $${\widetilde{U}}_{x}=\frac{\frac{1}{N}{\sum }_{n=1}^{N}{\int }_{V}{U}_{x}(x,{t}_{{\phi }}+(\text{n}-1)\text{T})dV}{{U}_{x, max}}\cdot 100 [\text{\%}],$$

17 $${\widetilde{U}}_{tke}=\frac{\frac{1}{N}{\sum }_{n=1}^{N}{\int }_{V}{U}_{tke}(x,{t}_{{\phi }}+(\text{n}-1)\text{T})dV}{{tke}_{max}}\cdot 100 [\text{\%}].$$

Previous studies showed that 50 samples were sufficient to obtain ± 5% convergence in the whole domain [26]. By increasing the number of samples, the mean velocity and the mean velocity fluctuations tend to converge in a smaller interval. For the chosen number of samples $(N  =  200)$± 2% convergence was obtained in the whole domain for all phases.

## RESULTS

### Hemodynamic Parameters

The aortic pressures and the flow rates matched the prescribed physiological values and varied only little between different experiments. The flow rates measured with the clamp-on flow meter were systematically higher (<7%) than the flow rates obtained from PIV. The mean transvalvular pressure gradients for the two mechanical valves were similar for low $CO$, while it was significantly larger for the Perimount. At normal $CO$, the pressure drop increased for the Perimount and the On-X while no increase was observed for the TRIFLO. At elevated $CO$, the pressure drop increased for all valves. However, it remained significantly lower for the TRIFLO valve. These differences in the pressure gradients were also reflected in the $EOA$ which was larger for TRIFLO at normal and elevated $CO$. The normalized uncertainties for the velocities and the turbulent kinetic energy, computed according to [10] and [11], were of the same order of magnitude in all experiments with values of less than 1% for all parameters. Results are summarized in Table 3.

**Table 3:** Flow values for each cardiac output and valve, low, normal, and elevated $CO$. In parenthesis, mean standard deviation over 60 consecutive cycles.

	$CO$	Transvalvular pressure gradient [mmHg]	Flow (Flowmeter) [l/min]	Flow (PIV) [l/min]	Mean systolic flow (PIV) [l/min]	EOA [cm^2^]
On-X	Low	5.53 (0.19)	3.09 (0.02)	2.97	10.08	1.52
	Normal	9.98 (0.67)	5.03 (0.03)	4.90	16.88	1.88
	Elevated	14.96 (0.56)	7.09 (0.04)	6.90	23.55	2.16
TRIFLO	Low	5.57 (0.08)	3.11 (0.02)	3.02	9.74	1.47
	Normal	5.41 (1.42)	5.15 (0.03)	4.96	16.89	2.56
	Elevated	9.14 (0.41)	7.29 (0.03)	6.89	23.49	2.75
Perimount	Low	9.92 (0.34)	3.12(0.02)	2.92	9.58	1.07
	Normal	12.86 (0.78)	5.22 (0.03)	4.91	16.55	1.63
	Elevated	15.68 (1.50)	7.29 (0.03)	7.22	24.12	2.16

### Velocity Field

**Fig. 4:**
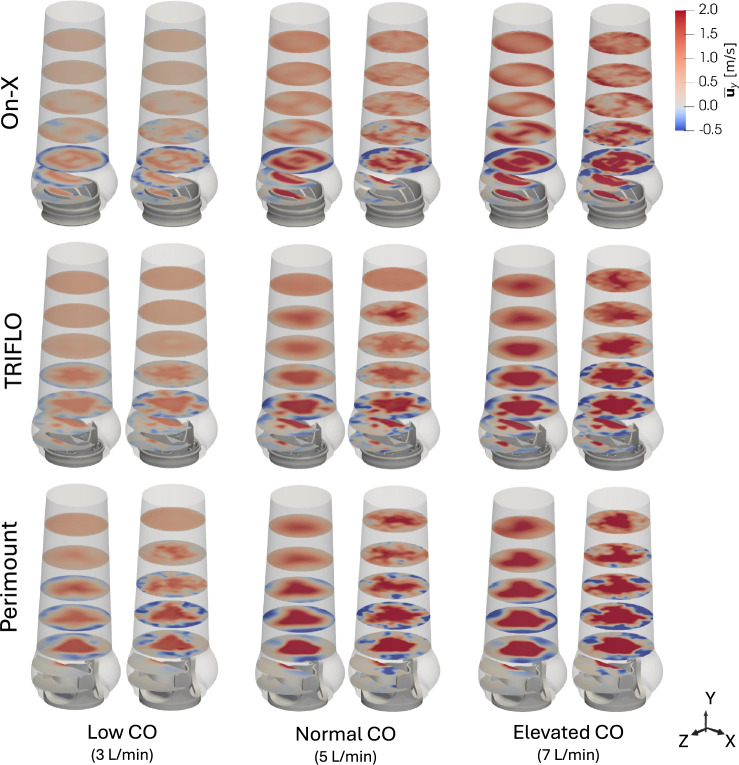
Mean (left) and instantaneous (right) streamwise velocities at peak systole ${t_{\phi}} = 130{\text{ms}}$

Figure 4 shows mean and representative example of instantaneous streamwise velocity fields at peak systole for all nine experiments. Visualization of additional temporal instances for mean streamwise velocity can be found in supplementary material. Each valve showed distinct flow patterns determined by their individual shape. These flow patterns persisted for all three flow conditions. The increase of $CO$ resulted in spatially extended influence of the valve design on the flow in the ascending aorta.

The Perimount was characterized by a single central systolic jet with a triangular-shaped cross section aligned with the valve posts. For increasing $CO$, the triangular shape of the central jet changed toward a more circular shape with larger area. Systolic retrograde flow along the aortic wall was observed in the surroundings of the central jet.

The TRIFLO was characterized by one central systolic jet of triangular shape and three small side jets in the sinus portions. In contrast to the Perimount, three distinct regions of retrograde flow along the aortic wall were located between the side jets and aligned with the valve posts (Figure 4).

Finally, the On-X was characterized by two semi-circular jets encompassing one smaller central jet. The systolic retrograde flow was concentrated in two regions along the aortic wall in the space between the two outer jets.

Further downstream in the ascending aorta, the flow fields for the TRIFLO and the Perimount showed central jets without retrograde flow, while semi-circular jets with high velocity near the wall and low velocities in the center persisted for the On-X.

Table 4 shows the mean peak velocities for all experiments. They were higher for the Perimount in all configurations compared to the mechanical valves, whereas TRIFLO showed the lowest values.

**Table 4:** Mean peak velocity in the aortic domain. For each output and valve, low, normal, and elevated $CO$ are shown, from top to bottom.

	Mean peak systolic v_max_ [m/s]	% Difference with respect to the Perimount
On-X	1.26	-31.5 %
	2.11	-19.8 %
	3.04	-10.5 %
TRIFLO	1.24	-32.6 %
	2.13	-24.0 %
	2.76	-18.8 %
Perimount	1.84	-
	2.63	-
	3.40	-

### Turbulent Kinetic Energy

**Fig. 5:**
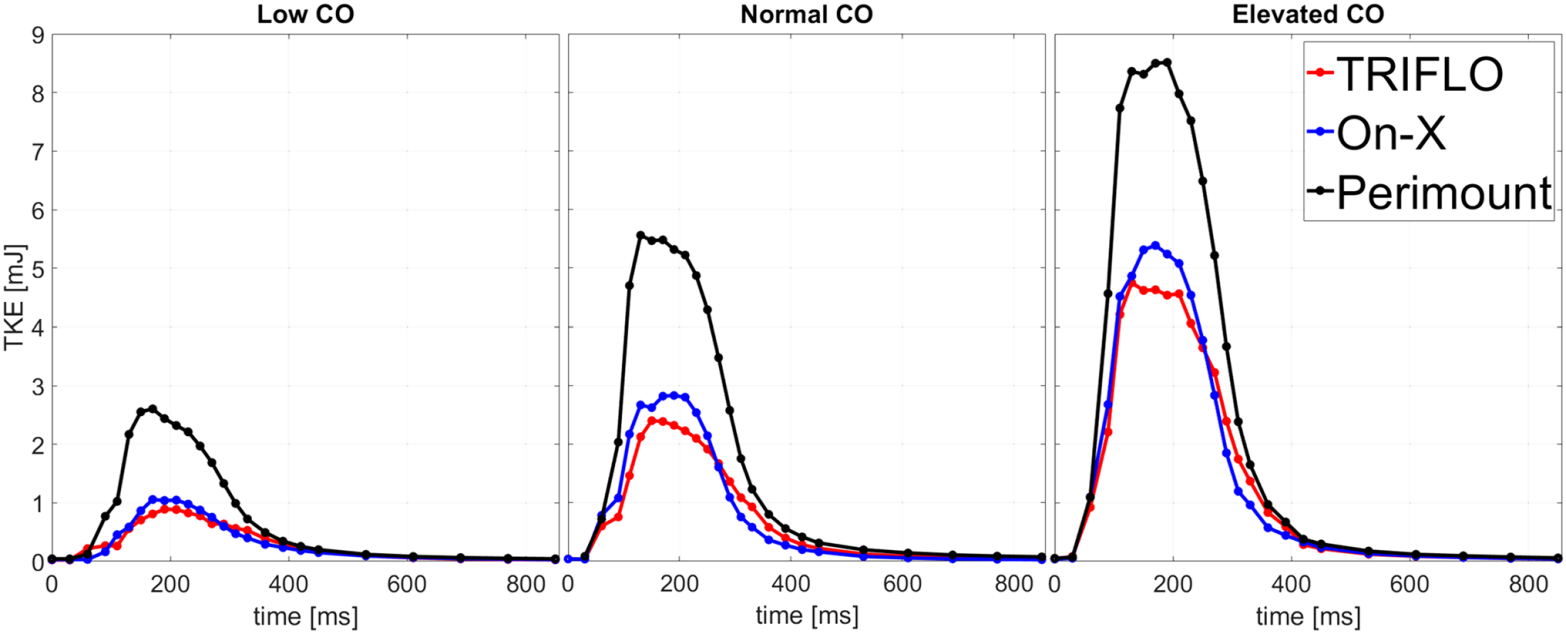
Comparison of $TKE$ for Perimount, On-X, and TRIFLO valves at low $CO$ (left), normal $CO$ (center), and elevated $CO$ (right).

The retrograde and antegrade flow domains in the ascending aorta were separated by shear layers. In these shear layers, the velocity fluctuations ${\nu^\prime}$ were dominant while they were nearly zero in the cores of the central jets of the Perimount and TRIFLO. These fluctuations resulted in elevated levels of turbulent kinetic energy which were high during systole and generally increased with increasing $CO$ (Figure 5). The TRIFLO showed the lowest systolic $TKE$ with slower decay during diastole in all configurations. Compared to the peak systolic $TKE$ for the Perimount, peak values for at low, normal, and elevated $CO$ were − 59.6%, − 49.5%, and − 36.1% for the On-X and − 65.8%, − 56.8%, and − 44.2% for the TRIFLO.

**Fig. 6:**
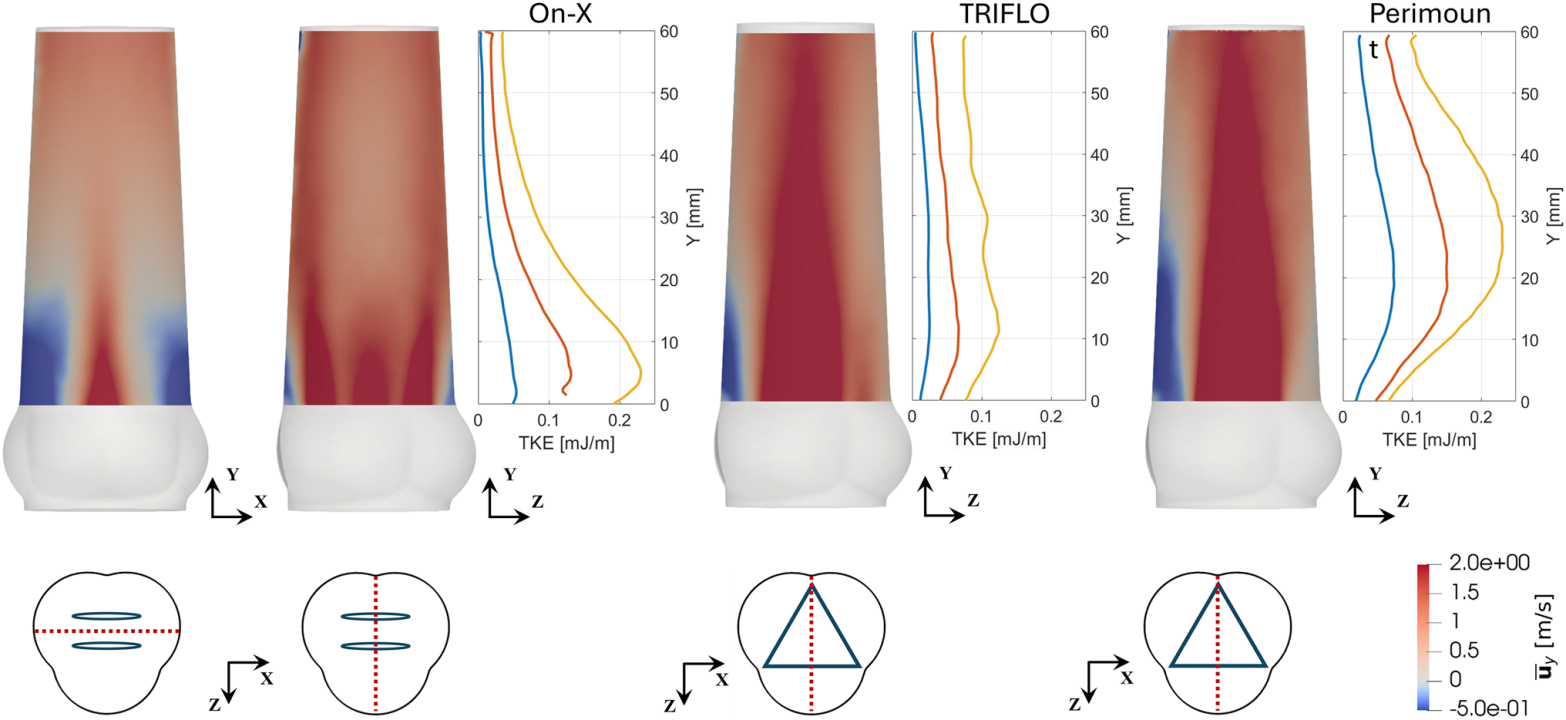
Planar $TKE$ shown together with mean streamwise velocity fields. The mean streamwise velocity at peak systole (elevated $CO$, ${t_{{\phi }}} = 130{\text{ms}}$) is computed along the axis of symmetry on the $yz$-plane for elevated $CO$. An additional plane shows the $xz$-plane for the On-X as the symmetry is present over 180° and not over 120° as in TRIFLO and Perimount. The vertical axis indicates the distance from the STJ.

The distribution of the planar $TKE$ along the axial direction revealed differences in the streamwise location of the peak values (Figure 6). For the Perimount, the peak planar $TKE$ is reached further downstream than for the TRIFLO and the On-X. The On-X reaches peak planar $TKE$ values already very close to the STJ. At the same time, the planar $TKE$ dissipated much faster in downstream direction than for the other valves, which showed higher planar $TKE$ values at the end of the measurement domain for all hemodynamic configurations.

The Kolmogorov scale $\eta$ showed decreasing size of the smallest turbulent structures with increasing cardiac output. Consistently with previous results, the minimum values of $\eta$ were found in the shear layer at peak systole [26]. The smallest structures were identified for the Perimount, followed by On-X and TRIFLO and were estimated to be between 40 and 90μm.

## Discussion

Quantitative comparison between different studies on heart valve protheses remains challenging due to differences in testing conditions and measurement methods [16]. The presented study aimed to close this gap by characterizing different types of surgical heart valve replacements in a systematic and consistent way. Moreover, to the authors’ best knowledge, this is the first study to present experimental results of reconstructed 3D flow fields at different hemodynamic conditions.

The heart valves were tested in a pulse replicator at 3, 5, and 7 l/min under hypotensive (40/60mmHg), normotensive (80/120mmHg), and moderate hypertensive (105/170mmHg) pressure conditions, respectively. The three-dimensional flow fields were reconstructed by means of Tomo-PIV at different phases of the cardiac cycle.

### Hemodynamic Parameters and Flow Topology

All experiments in this study were carried out in the same experimental setup. The conditions for the different valves were almost identical despite small variations in mean flow rate and pressures. This allows for a direct comparison of the hemodynamic parameters and flow fields of the three valves at three different $COs$.

Density and kinematic viscosity of the testing fluid were higher than for blood [34, 35]. Nevertheless, the Reynolds ($Re\approx 3500-7000)$ and Womersley $\left(Wo\approx 13\right)$ numbers remained in the physiological range reported in the literature, ensuring dynamic similarity to reality and other experiments [36–39].

Studies on patients undergoing surgical valve replacement reported mean transvalvular gradients of 13.4 ± 5.7 and 10.3 ± 3mmHg after 21mm Perimount and On-X valve implantation, respectively [40, 41]. These values are in good agreement with the present measurements obtained at normal $CO$ of 12.86 ± 0.78 (Perimount) and 9.98 ± 0.02mmHg (On-X).

The transvalvular pressure gradient increased with increasing $CO$ for all valves. The On-X had the sharpest relative increase, while the Perimount showed the highest values at all conditions. For all valves, the $EOA$ was highest for the elevated $CO$ which indicates that the hydraulic resistance decreased for higher $CO$. TRIFLO had the lowest pressure gradient and the highest $EOA$ for all hemodynamic conditions, except for low $CO$ where results were comparable to the On-X. This suggests that TRIFLO valve provides the best global hemodynamic performance. While the hemodynamic performance of the Perimount was weakest at low and normal $CO$, it improved with increasing $CO$ and was comparable to the On-X for elevated $CO$.

For increasing $CO$, larger and more distinct areas of forward flow (e.g., central jets in Perimount and TRIFLO) were observed for all valves, and the footprint of the valve design on the flow fields extended farther into the ascending aorta. For Perimount, this may be partly related to an increased opening of the valve with higher $CO$, whereas we attribute this effect for the mechanical valves mostly to higher flow inertia due to higher $CO$. This higher inertia is probably also accountable for the later reattachment of the central jets at the aortic wall (in Perimount and TRIFLO) increasing the distal extent of the regions of retrograde flow along the aortic wall.

Although TRIFLO and Perimount exhibited similar flow patterns, there were some distinct differences: The side jets observed only for the TRIFLO were previously described as beneficial for the washout of the sinuses, as they may reduce the possibility of flow stagnation in this region [42]. Systolic retrograde flows were observed in the surroundings of the central jet for the Perimount and above the posts of the valve for the TRIFLO. Finally, the On-X was characterized by a central jet surrounded by two semi-circular jets, leaving space for retrograde flow along the aortic wall, in the space between the two outer jets. Peak velocities located closer to the aortic wall exposed the wall and the adjacent blood flow to higher levels of shear stress compared to flow configuration with peak velocity in the center.

The TRIFLO valve showed lower peak velocities, lower transvalvular pressure gradients, higher $EOAs$, and lower $TKE$ than the other valves. In contrast, the Perimount showed the highest values.

### Turbulent Kinetic Energy

The Perimount showed the highest $TKE$ for all $CO$. This was somewhat unexpected, but it is in line with the transvalvular pressure gradients (Table 3) that were obtained independently with pressure sensors. We attribute this mainly to the smaller geometric orifice area of the Perimount (compared to the On-X and TRIFLO): although all valves had the same labeling size of 21mm, the design of the On-X and TRIFLO allowed for larger orifice areas than the Perimount. This reduced opening can also explain the increased peak velocities of the Perimount which is associated with a larger Reynolds number of the central jet, the elevated $TKE$, and the higher transvalvular pressure gradient.

The distribution of planar $TKE$ along the central axis can provide further insight: At the beginning of the systole, the turbulence in the aorta was not yet fully developed and pulse-to-pulse fluctuations may have contributed significantly and spuriously to the $TKE$. These pulse-to-pulse fluctuations are associated with the front of the aortic jet which issues into the ascending aorta and includes the starting vortex. This could lead to spurious fluctuations in the Reynolds decomposed flow field because the jet front and starting vortex could be at slightly different positions in different pulses. Once the jet is fully developed at peak systole, the streamwise gradual increase of planar $TKE$ is well captured for TRIFLO and Perimount as seen in Figure 6. In contrast, the streamwise peaks of planar $TKE$ for the On-X are located very close to the valve. Accordingly, the plane closest to the STJ exhibits much higher values for planar $TKE$ compared to TRIFLO and Perimount suggesting that high values of planar $TKE$ could also exist between the valve and the STJ which was not captured by our experiments. Therefore, the $TKE$ of the On-X is probably somewhat underestimated (due to the missing contribution proximal to the STJ) which could in part explain the higher integral values of $TKE$ for Perimount compared to BHMV.

A combined interpretation of the results from flow velocities and planar $TKE$ provides a more comprehensive comparison of the different valve types. Although the $TKE$ is significantly higher for the Perimount, the distribution of planar $TKE$ shows a more differentiated picture. At low $CO$, peak values of planar $TKE$ of the Perimount are higher than for the other two valves. For increasing $CO$, the amplitudes of the planar $TKE$ become comparable for the Perimount and the On-X. Of particular interest is the location of these peaks: for the Perimount, they are 20–30mm downstream of the STJ which agrees well with the computational predictions in [8], whereas they are near the STJ for the On-X. This is in line with the higher thrombogenic risk associated with the On-X. The high $TKE$ peak close to the STJ exposes blood platelets to elevated shear rates already near the sinus portions, where blood flow may be subject to stagnation promoting coagulation [43].

## Limitations

Integrating a mock left heart circuit with a complex Tomo-PIV system posed significant challenges requiring some simplifications. First, the aortic phantom represented an idealized geometry which did not replicate the coronary ostia. As the coronary perfusion occurs mainly during diastole accounting for approximately 5% of the cardiac output, small retrograde flow would be expected in this period [44].

Moreover, the semi-rigid model neither featured the compliance of the aortic wall and the aortic arch. The influence of wall stiffness was investigated in the work of Jahren et al. on an analog mock circuit, showing no influence of this parameter on the flow rate and only small increase in pressure gradient with increasing wall rigidity [29].

Lacking the aortic arch, the experimental setup did not show any secondary flows due to curvature which could have altered the impingement of the turbulent wake in the aorta. However, the present analysis was performed within 2–3 aortic diameters from the aortic annulus, where the effect of curvature-generated secondary flows could be considered small. Moreover, it should be emphasized again that this study did not intend to evaluate the clinical outcome of different valve prostheses but to provide a systematic comparison of different valve types at different hemodynamic conditions.

Additional considerations regarding the osmotic concentration of the testing fluid are necessary when comparing mechanical to biological valves. Elevated concentrations of NaCl (compared to blood) could affect the mechanical properties of the biological valve, potentially leading to performance variations over time. Despite the lack of any visible degradation and the short time, the valve was immersed in the fluid (<2 h per experiment), the effect of high salt concentrations on leaflet mechanics should be further investigated.

Because of the limited spatial resolution, it was not possible to resolve the turbulent structures to the smallest scales and to accurately compute instantaneous viscous shear stresses. Therefore, the reported Kolmogorov length scales are probably overestimated.

## Supplementary Information

Below is the link to the electronic supplementary material.

Supplementary file 1 (PDF 2245 KB)

## References

[1] Brown, J. M., S. M. O’Brien, C. Wu, J. A. H. Sikora, B. P. Griffith, and J. S. Gammie. Isolated aortic valve replacement in North America comprising 108,687 patients in 10 years: Changes in risks, valve types, and outcomes in the Society of Thoracic Surgeons National Database. *J. Thorac. Cardiovasc. Surg.* 137(1):82–90, 2009. 19154908 10.1016/j.jtcvs.2008.08.015

[2] Vahanian, A., F. Beyersdorf, F. Praz, M. Milojevic, S. Baldus, J. Bauersachs, et al. 2021 ESC/EACTS Guidelines for the management of valvular heart disease: Developed by the Task Force for the management of valvular heart disease of the European Society of Cardiology (ESC) and the European Association for Cardio-Thoracic Surgery (EACTS). *Rev. Esp. Cardiol. Engl. Ed.* 75(6):524, 2022. 35636831 10.1016/j.rec.2022.05.006

[3] Carlin, S., and J. Eikelboom. Advances in anticoagulation: patients with bioprosthetic heart valves. *Cardiovasc. Res.* 118(3):e26–e28, 2022. 35024787 10.1093/cvr/cvab360

[4] Jamieson, W. R. E., M. T. Janusz, L. H. Burr, H. Ling, R. T. Miyagishima, and E. Germann. Carpentier-Edwards supraannular porcine bioprosthesis: second-generation prosthesis in aortic valve replacement. *Ann. Thorac. Surg.* 71(5):S224–S227, 2001. 11388191 10.1016/s0003-4975(01)02549-8

[5] MacIsaac, S., I. H. Jaffer, E. P. Belley-Côté, G. R. McClure, J. W. Eikelboom, and R. P. Whitlock. How did we get here?: A historical review and critical analysis of anticoagulation therapy following mechanical valve replacement. *Circulation*. 140(23):1933–1942, 2019. 31790297 10.1161/CIRCULATIONAHA.119.041105

[6] Hedayat, M., and I. Borazjani. Comparison of platelet activation through hinge vs bulk flow in bileaflet mechanical heart valves. *J. Biomech.* 83:280–290, 2019. 30579576 10.1016/j.jbiomech.2018.12.003

[7] Borazjani, I. Fluid–structure interaction, immersed boundary-finite element method simulations of bio-prosthetic heart valves. *Comput. Methods Appl. Mech. Eng.* 257:103–116, 2013.

[8] Becsek, B., L. Pietrasanta, and D. Obrist. Turbulent systolic flow downstream of a bioprosthetic aortic valve: velocity spectra, wall shear stresses, and turbulent dissipation rates. *Front Physiol.* 29(11):577188, 2020. 10.3389/fphys.2020.577188 PMC7550765 33117194

[9] Hasler, D., A. Landolt, and D. Obrist. Tomographic PIV behind a prosthetic heart valve. *Exp Fluids.* 57(5):80, 2016.

[10] (EN) ISO_5840-1_2021 Cardiovascular implants- Cardiac valve prostheses-Part 1 General Requirements.PDF.

[11] (EN) ISO_5840-2_2021 Cardiovascular implants- Cardiac valve prostheses-Part 2 Surgically implanted heart valve substitutes.PDF.

[12] (EN) ISO_5840-3_2021 Cardiovascular implants-Cardiac valve prostheses Part 3 Heart Valve substitutes implanted by transcatheter techniques.PDF.

[13] Doenst, T., P. A. Amorim, N. Al-Alam, S. Lehmann, C. Mukherjee, and G. Faerber. Where is the common sense in aortic valve replacement? A review of hemodynamics and sizing of stented tissue valves. *J. Thorac. Cardiovasc. Surg.* 142(5):1180–1187, 2011. 21703637 10.1016/j.jtcvs.2011.05.007

[14] Kytö, V., J. Sipilä, E. Ahtela, P. Rautava, and J. Gunn. Mechanical versus biologic prostheses for surgical aortic valve replacement in patients aged 50 to 70. *Ann. Thorac. Surg.* 110(1):102–110, 2020. 31786289 10.1016/j.athoracsur.2019.10.027

[15] Pibarot, P., and J. G. Dumesnil. Prosthetic heart valves: selection of the optimal prosthesis and long-term management. *Circulation*. 119(7):1034–1048, 2009. 19237674 10.1161/CIRCULATIONAHA.108.778886

[16] Wu, C., N. Saikrishnan, A. J. Chalekian, R. Fraser, O. Ieropoli, S. M. Retta, et al. In-vitro pulsatile flow testing of prosthetic heart valves: a round-robin study by the ISO cardiac valves working group. *Cardiovasc. Eng. Technol.* 10(3):397–422, 2019. 31240664 10.1007/s13239-019-00422-5

[17] Marquez, S., R. T. Hon, and A. P. Yoganathan. Comparative hydrodynamic evaluation of bioprosthetic heart valves. *J. Heart Valve Dis.* 10(6):802–811, 2001. 11767190

[18] Gerosa, G., V. Tarzia, G. Rizzoli, and T. Bottio. Small aortic annulus: the hydrodynamic performances of 5 commercially available tissue valves. *J. Thorac. Cardiovasc. Surg.* 131(5):1058–1064, 2006. 16678590 10.1016/j.jtcvs.2005.12.034

[19] Sturla, F., F. Piatti, M. Jaworek, F. Lucherini, F. R. Pluchinotta, S. V. Siryk, et al. 4D Flow MRI hemodynamic benchmarking of surgical bioprosthetic valves. *Magn. Reson. Imaging*. 68:18–29, 2020. 31981709 10.1016/j.mri.2020.01.006

[20] Bottio, T., L. Caprili, D. Casarotto, and G. Gerosa. Small aortic annulus: the hydrodynamic performances of 5 commercially available bileaflet mechanical valves. *J. Thorac. Cardiovasc. Surg.* 128(3):457–462, 2004. 15354108 10.1016/j.jtcvs.2004.03.021

[21] Bottio, T., V. Tarzia, G. Rizzoli, and G. Gerosa. In-vitro testing of three totally supra-annular bileaflet mechanical valves: hydrodynamics in the Sheffield pulse duplicator. *J. Heart Valve Dis.* 17(2):222–226, 2008. 18512495

[22] Hasler, D., and D. Obrist. Three-dimensional flow structures past a bio-prosthetic valve in an in-vitro model of the aortic root. Borazjani I, editor. *PLoS ONE*. 13(3):e019384, 2018. 10.1371/journal.pone.0194384 PMC5856406 29547668

[23] Elsinga, G. E., F. Scarano, B. Wieneke, and B. W. Van Oudheusden. Tomographic particle image velocimetry. *Exp. Fluids*. 41(6):933–947, 2006.

[24] Zeugin, T., F. B. Coulter, U. Gülan, A. R. Studart, and M. Holzner. In vitro investigation of the blood flow downstream of a 3D-printed aortic valve. *Sci. Rep.* 18(14):1572, 2024. 10.1038/s41598-024-51676-6 PMC10796383 38238358

[25] Gülan, U., H. Appa, P. Corso, C. Templin, D. Bezuidenhout, P. Zilla, et al. Performance analysis of the transcatheter aortic valve implantation on blood flow hemodynamics: An optical imaging-based in vitro study. *Artif. Org.* 43(10):E282–E293, 2019. 10.1111/aor.13504 31140632

[26] Pietrasanta, L., S. Zheng, D. De Marinis, D. Hasler, and D. Obrist. Characterization of turbulent flow behind a transcatheter aortic valve in different implantation positions. *Front Cardiovasc. Med.* 13(8):804565, 2022. 10.3389/fcvm.2021.804565 PMC8794584 35097022

[27] Chen, H., and L. P. Dasi. An in-vitro study of the flow past a transcatheter aortic valve using time-resolved 3D particle tracking. *Ann. Biomed. Eng.* 51(7):1449–1460, 2023. 36705865 10.1007/s10439-023-03147-8

[28] Swanson, M., and R. E. Clark. Dimensions and geometric relationships of the human aortic valve as a function of pressure. *Circ. Res.* 35(6):871–882, 1974. 4471354 10.1161/01.res.35.6.871

[29] Jahren, S. E., B. M. Winkler, P. P. Heinisch, J. Wirz, T. Carrel, and D. Obrist. Aortic root stiffness affects the kinematics of bioprosthetic aortic valves. *Interact. Cardiovasc. Thorac. Surg.* 24(2):173–180, 2017. 27680580 10.1093/icvts/ivw284

[30] Pope, S. B. Turbulent Flows. Cambridge: Cambridge University Press, p. 810, 2000.

[31] Raghav, V., S. Sastry, and N. Saikrishnan. Experimental assessment of flow fields associated with heart valve prostheses using particle image velocimetry (PIV): recommendations for best practices. *Cardiovasc. Eng. Technol.* 9(3):273–287, 2018. 29532332 10.1007/s13239-018-0348-z

[32] Wieneke, B. Volume self-calibration for 3D particle image velocimetry. *Exp Fluids.* 45(4):549–556, 2008.

[33] Sciacchitano, A., and B. Wieneke. PIV uncertainty propagation. *Meas Sci Technol.* 27(8):084006, 2016. 10.1088/0957-0233/27/8/084012 PMC4972504 27499587

[34] Rosencranz, R., and S. A. Bogen. Clinical laboratory measurement of serum, plasma, and blood viscosity. *Pathol. Patterns Rev.* 125(1):78–86, 2006. 10.1309/FFF7U8RRPK26VAPY 16830959

[35] Rosenson, R. S., A. McCormick, and E. F. Uretz. Distribution of blood viscosity values and biochemical correlates in healthy adults. *Clin. Chem.* 42(8):1189–1195, 1996. 8697575

[36] Bauer, A., S. Wegt, M. Bopp, S. Jakirlić, C. Tropea, A. Krafft, et al. Comparison of wall shear stress estimates obtained by laser Doppler velocimetry, magnetic resonance imaging and numerical simulations. *Exp. Fluids*. 18:60, 2019.

[37] Stein, P. D., and H. N. Sabbah. Turbulent blood flow in the ascending aorta of humans with normal and diseased aortic valves. *Circ. Res.* 39(1):58–65, 1976. 776437 10.1161/01.res.39.1.58

[38] Doutel, E., F. Galindo-Rosales, and L. Campo-Deaño. Hemodynamics challenges for the navigation of medical microbots for the treatment of CVDs. *Materials.* 2(14):7402, 2021. 10.3390/ma14237402 PMC8658690 34885556

[39] San, O., and A. Staples. An improved model for reduced-order physiological fluid flows. *J. Mech. Med. Biol.* 19:12, 2012.

[40] Dalmau, M. J., J. María González-Santos, J. López-Rodríguez, M. Bueno, and A. Arribas. The Carpentier-Edwards Perimount Magna aortic xenograft: a new design with an improved hemodynamic performance. *Interact Cardiovasc. Thorac. Surg.* 5(3):263–267, 2006. 17670564 10.1510/icvts.2005.120352

[41] Ozyurda, U., A. R. Akar, O. Uymaz, M. Oguz, M. Ozkan, C. Yildirim, et al. Early clinical experience with the On-X prosthetic heart valve. *Interact Cardiovasc. Thorac. Surg.* 1(4):588–594, 2006. 10.1510/icvts.2005.114843 17670488

[42] Pietrasanta L. Experimental Investigation of the Three-dimensional Flow Field of Novel Aortic Valve Prosthesis Designs [Doctoral thesis]. [Bern]: University of Bern; 2022.

[43] Dasi, L. P., H. A. Simon, P. Sucosky, and A. P. Yoganathan. Fluid mechanics of artificial heart valves. *Clin. Exp. Pharmacol. Physiol.* 36(2):225–237, 2009. 19220329 10.1111/j.1440-1681.2008.05099.x PMC2752693

[44] Ramanathan, T., and H. Skinner. Coronary blood flow. *Contin. Educ. Anaesth Crit. Care Pain.* 5(2):61–64, 2005.

