# Supplementary figures and images for: Comparison of Hemodynamic Performance, Three-Dimensional Flow Fields, and Turbulence Levels for Three Different Heart Valves at Three Different Hemodynamic Conditions
Source: Ann Biomed Eng. 2024 Sep 17;52(12):3196–207. doi: 10.1007/s10439-024-03584-z (PMC11561026; doi:10.1007/s10439-024-03584-z)

On-X

3 L/min

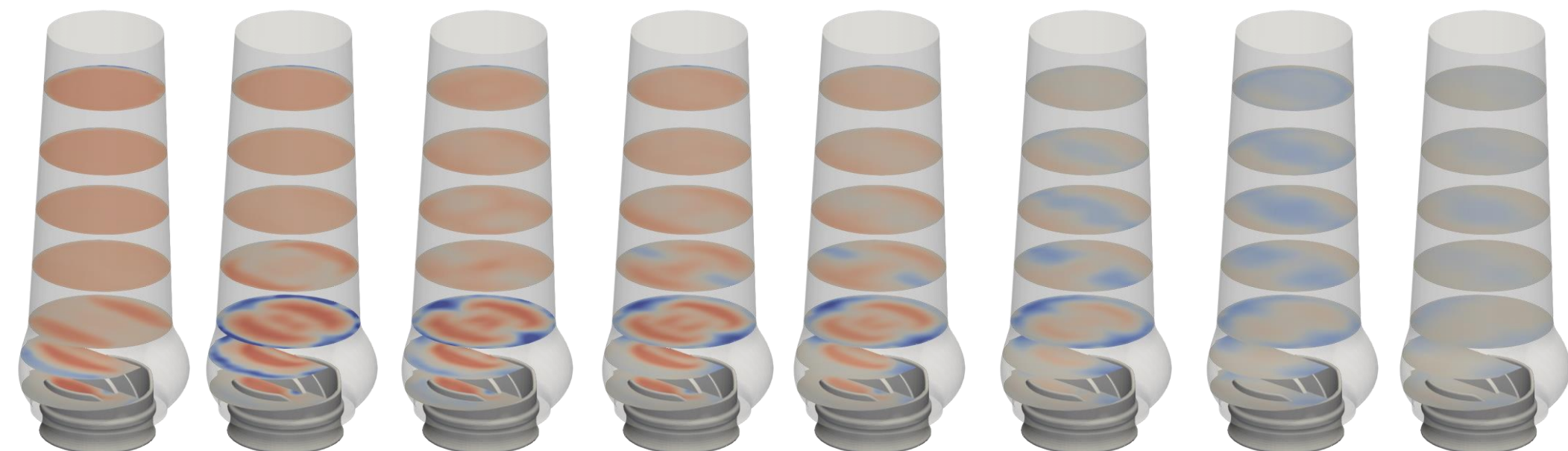

5 L/min

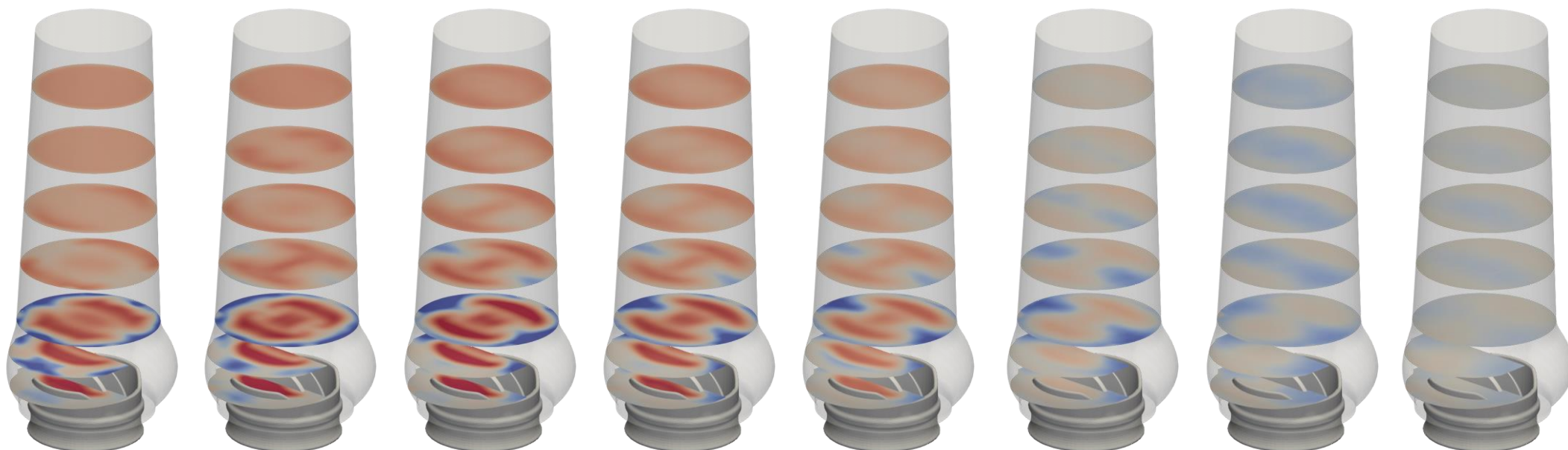

7 L/min

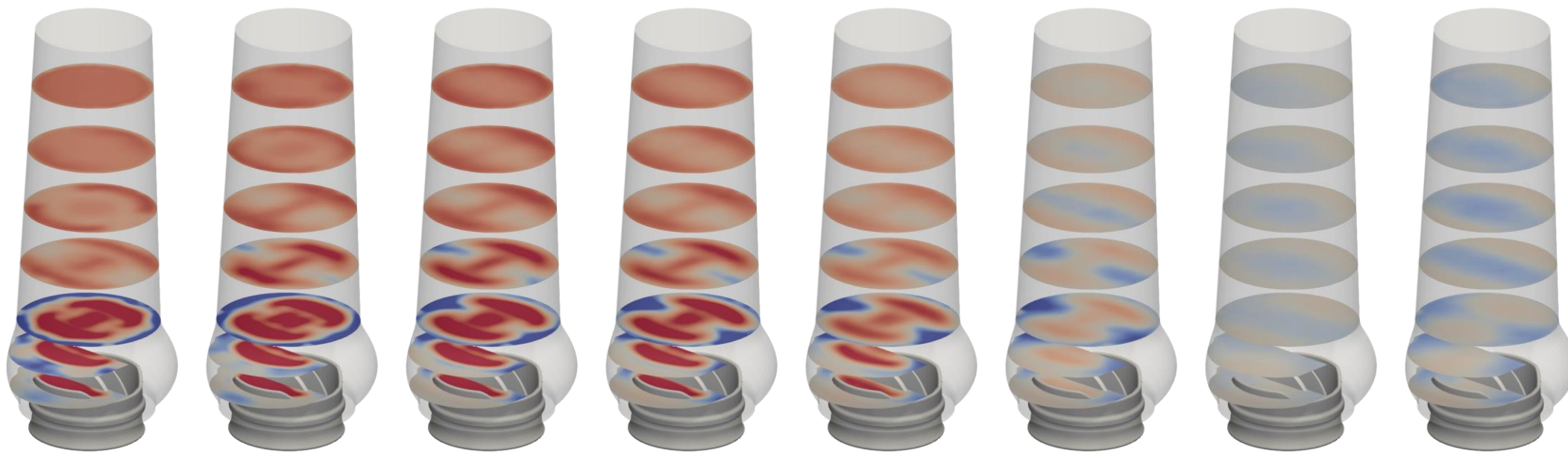

90

110

170

210

250

290

330

390

Time (ms)

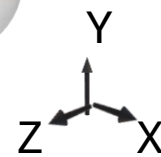

$\bar{u}_y$  [m/s]

2.0  
1.5  
1.0  
0.5  
0.0  
-0.5

# TRIFLO

3 L/min

5 L/min

7 L/min

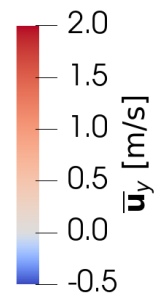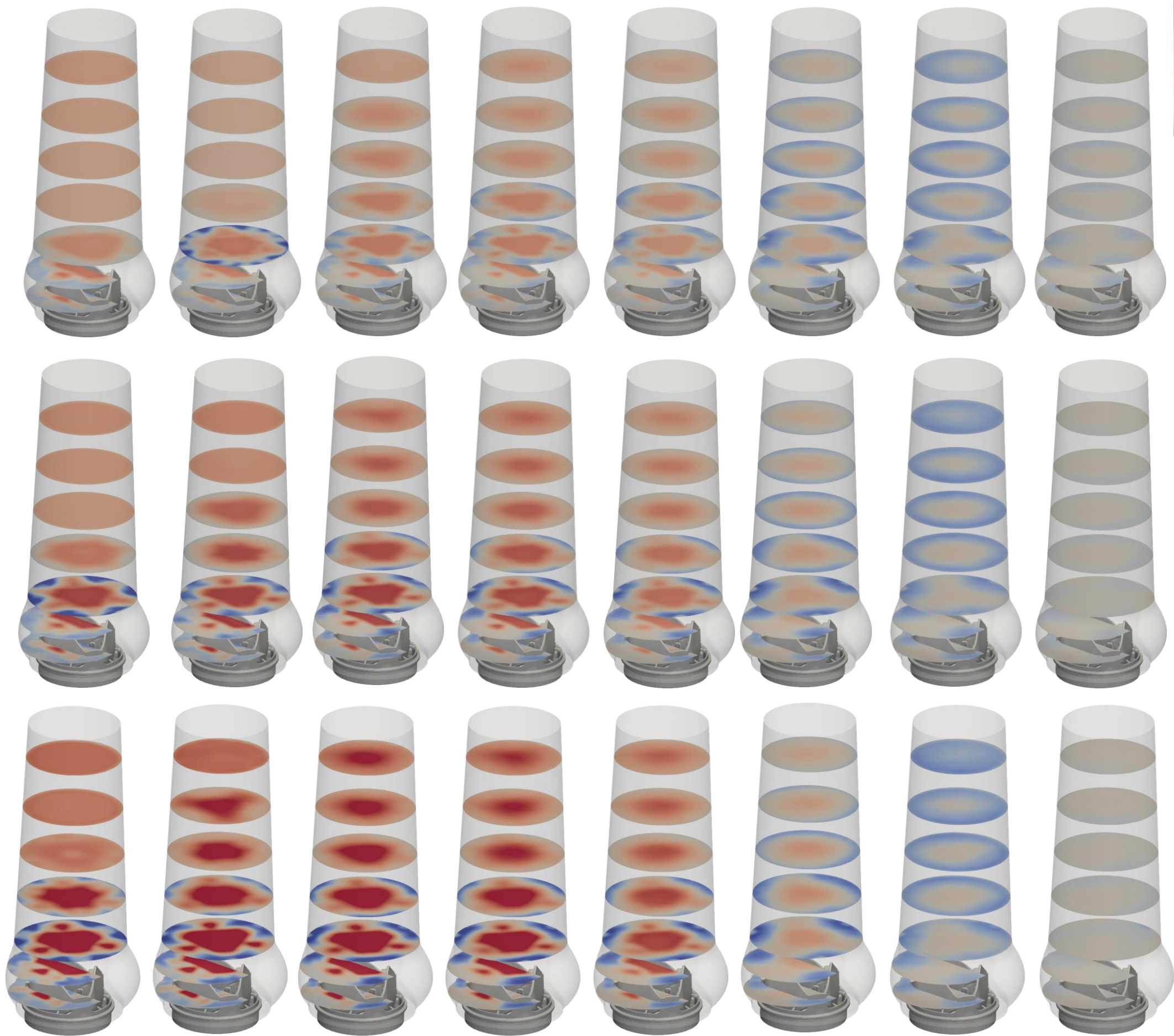

90

110

170

210

250

290

330

390

Time (ms)

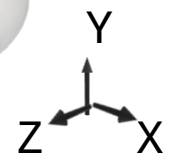

# Perimount

3 L/min

5 L/min

7 L/min

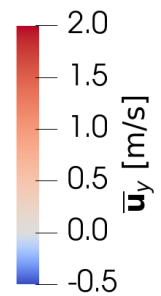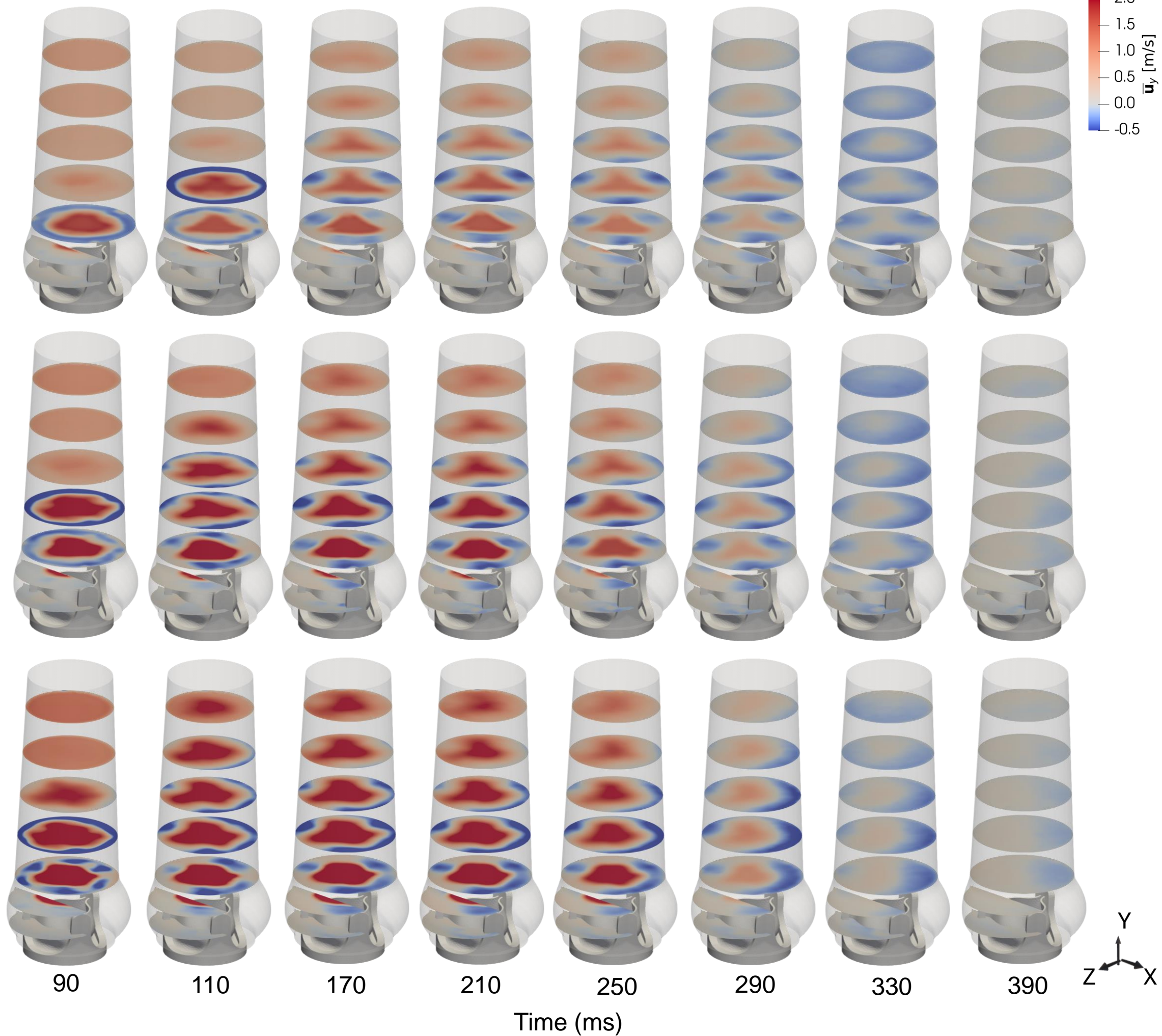

Supplement: Supplementary file 1 — (PDF 2245 KB) [file 10439_2024_3584_MOESM1_ESM.pdf]
